# Using lanthanoid complexes to phase large macromolecular assemblies

**DOI:** 10.1107/S0909049510036824

**Published:** 2010-11-05

**Authors:** Romain Talon, Richard Kahn, M. Asunción Durá, Olivier Maury, Frédéric M. D. Vellieux, Bruno Franzetti, Eric Girard

**Affiliations:** a CEA, IBS, F-38054 Grenoble, France; b CNRS, UMR5075, F-38027 Grenoble, France; c Université Joseph Fourier, F-38000 Grenoble, France; d Université de Lyon, Ecole Normale Supérieure de Lyon, Laboratoire de Chimie, UMR CNRS 5182, 46 Allée d’Italie, 69364 Lyon Cedex 07, France

**Keywords:** lanthanoid complexes, experimental phasing, large macromolecular assemblies, TET aminopeptidase

## Abstract

Lanthanoid ions exhibit extremely large anomalous X-ray scattering at their *L*_III_ absorption edge. They are thus well suited for anomalous diffraction experiments. A novel class of lanthanoid complexes has been developed that combines the physical properties of lanthanoid atoms with functional chemical groups that allow non-covalent binding to proteins. Two structures of large multimeric proteins have already been determined by using such complexes. Here the use of the luminescent europium tris-dipicolinate complex [Eu(DPA)_3_]^3−^ to solve the low-resolution structure of a 444 kDa homo­dodecameric aminopeptidase, called PhTET1-12s from the archaea *Pyrococcus horikoshii*, is reported. Surprisingly, considering the low resolution of the data, the experimental electron density map is very well defined. Experimental phases obtained by using the lanthanoid complex lead to maps displaying particular structural features usually observed in higher-resolution maps. Such complexes open a new way for solving the structure of large molecular assemblies, even with low-resolution data.

## Abstract 2

A lanthanoid complex, [Eu(DPA)_3_]^3−^, was used to obtain experimental phases at 4.0 Å resolution of PhTET1-12s, a large self-compartmentalized homo-dodecameric protease complex of 444 kDa.

## 1. Introduction

Even though most of the newly deposited structures in the Protein Data Bank (PDB) were solved by molecular replacement, experimental phasing remains essential for determining three-dimensional protein structures if only for solving structures with new folds or which significantly differ from any known model structure. Over the last ten years, methods based on anomalous scattering, namely the single-wavelength anomalous diffraction (SAD) and multiple-wavelength anomalous diffraction (MAD) methods, have replaced the traditional methods based on isomorphous replacement, thus becoming the methods of choice for solving *de novo* protein structures. Consequently, the preparation of effective heavy-atom derivatives displaying anomalous scattering has become a key point for *de novo* crystal structure determination. With the incorporation of selenium through the substitution of methionine residues by seleno-methionine (Hendrickson *et al.*, 1990 ▶; Doublie, 1997 ▶), and with the developments at third-generation synchrotron radiation sources, which allow weak anomalous signals from intrinsic scatterers to be used, the time-consuming preparation of heavy-atom derivatives has been facilitated.

However, the use of such procedures is not always possible, which revives the problem of incorporating effective anomalous scatterers into protein crystals. Therefore, we proposed to use lanthanoid complexes for preparing lanthanoid derivative crystals (Girard *et al.*, 2002 ▶). Lanthanoid ions, Ln^3+^, are well suited to anomalous diffraction experiments since they all exhibit a strong white line in their *L*_III_ absorption edge leading to extremely large anomalous contributions of almost 30 e^−^ for both *f*′ and *f*′′.

A way to assess the phasing power of lanthanides is to compare them with the most frequently used anomalous scatterer, *i.e.* selenium from seleno-methionine. For this purpose the Bijvoet ratio can be considered. We have shown (Girard, Stelter *et al.*, 2003 ▶) that the Bijvoet ratio can be expressed as
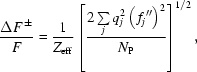
where 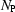 is the number of atoms of the protein of mean scattering factor 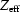, 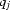 and 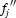 are the site occupancy and the imaginary part of the atomic scattering factor of the anomalous scatterer 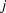, respectively. This formula clearly shows that, assuming fixed site occupancies, identical Bijvoet ratios are obtained for a protein that is four times larger when 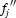 is doubled for each anomalous scatterer. Assuming that the diffraction data are collected at the respective absorption edge, 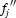 values are about 10 and 30 e^−^ for selenium and lanthanoid, respectively. This means that one fully occupied lanthanoid atom will allow a protein that is nine times larger, compared with a fully occupied Se atom, to be phased.

Hence, lanthanoids are good candidates for macromolecular structure determination based on the use of the anomalous signal. Lanthanoid ions were used in early MAD studies on calcium-binding proteins (Kahn *et al.*, 1985 ▶; Weis *et al.*, 1991 ▶) as they can substitute for Ca^2+^. Nagem *et al.* (2001 ▶) proposed to incorporate lanthanoid salts through the quick cryo-soak method, but soaking crystals in solutions containing lanthanoid salts often damages the crystals owing to the preferred nine-based coordination of lanthanoid ions. To overcome this problem, Purdy *et al.* (2002 ▶) proposed to use a covalent linkage between a lanthanoid complex featuring a saturated coordination sphere and the protein of interest through a thio-reactive functionality, and Silvaggi *et al.* (2007 ▶) proposed to use a double lanthanoid-binding tag.

Girard *et al.* (2002 ▶) proposed to use gadolinium complexes initially used as contrast agents for magnetic resonant imaging to incorporate lanthanoid ions into protein crystals. Seven different gadolinium complexes were studied (Girard, Stelter *et al.*, 2003 ▶). These complexes are made of a ligand that surrounds the lanthanoid ions as a cage, thus providing the majority of the coordination sphere of the ion. More recently, we have proposed to use complexes based on dipicolinate (DPA = pyridine-2,6-dicarboxylate) ligands, namely the lanthanoid tris-dipicolinate complex ions [Ln(DPA)_3_]^3−^, the Eu and Tb complexes being luminescent (D’Aléo *et al.*, 2007 ▶; Pompidor *et al.*, 2008 ▶). As previously mentioned, lanthanoid salts often damage protein crystals even at low concentration. The great advantage of using lanthanoid complexes comes from the fact that the interaction of the lanthanoid ions with the protein occurs through the ligand forming the complexes rather than direct interaction. The binding mode of the various used complexes to the protein turned out to depend on the nature of the ligand (Girard, Anelli *et al.*, 2003 ▶). These complexes can be introduced in protein crystals either by co-crystallization or soaking and can be used at rather high concentration (50 to 100 m*M*).

The technique of introducing lanthanoid ions into protein crystals by using lanthanoid complexes^1^ was successfully used to solve the structure of several proteins (Chaudhuri *et al.*, 2003 ▶; de Bono *et al.*, 2005 ▶; Hermoso *et al.*, 2005 ▶; Jeudy *et al.*, 2005 ▶; Márquez *et al.*, 2006 ▶; Gras *et al.*, 2007 ▶; Delfosse *et al.*, 2009 ▶; Molina *et al.*, 2009 ▶; Arnoux *et al.*, 2009 ▶; Pérez-Dorado, González *et al.*, 2010 ▶; Pérez-Dorado, Sanles *et al.*, 2010 ▶).

The lanthanoid complexes have also been used to solve structures of large macromolecular assemblies. The structure of a chimeric ornithine carbamoyl transferase, OTCase3630, a dodecamer of 450 kDa, was solved by using the SAD method (Girard, Stelter *et al.*, 2003 ▶). More recently, the structure of the *Pyrococcus abyssi* Pab87 protein, an archaeal member of a new self-compartmentalizing protease family forming a cubic-shaped octamer of 400 kDa, was determined at 2.2 Å resolution by the SAD method (Delfosse *et al.*, 2009 ▶).

Here, we report the use of the tris-dipicolinate complex to obtain experimental phases at low resolution on a large homo-dodecameric enzyme, PhTET1-12s, which is a tetrahedral aminopeptidase belonging to a new family of self-compartmentalized large protease complexes (Franzetti *et al.*, 2002 ▶). The TET peptidase was initially isolated from *Haloarcula marismortui* (Franzetti *et al.*, 2002 ▶). In the archae *Pyrococcus horikoshii*, three different open reading frames coding for TET-homologous proteins were identified. These were named PhTET1, 2 and 3. Their three-dimensional structures were determined (Franzetti *et al.*, 2002 ▶; Russo & Baumann, 2004 ▶; Borissenko & Groll, 2005 ▶; Schoehn *et al.*, 2006 ▶; Durá *et al.*, 2009 ▶). It has been shown (Schoehn *et al.*, 2006 ▶) that PhTET1 assembles as a tetrahedral dodecameric particle (called PhTET1-12s for the 444 kDa assembly made up of 12 sub­units) or as an octahedral tetracosameric edifice (called PhTET1-24s for the 888 kDa assembly made up of 24 sub­units).

Since the TET particles are highly symmetrical molecular edifices formed by a single type of subunit, they provide an excellent model for probing the phasing capacity of different lanthanoid complexes. Moreover, the currently available TET crystallographic structures do not permit detailed analyses of the particles interior. The polypeptide trafficking and the processing mechanisms by the TET particles remain therefore unclear. In this paper we show that low-resolution experimental phase obtained with tris-dipicolinate complex can provide novel structural information on the PhTET1-12s complex.

## 2. Materials and methods

### 2.1. Materials

The expression of TET1-12s from *P. horikoshii* in *Escherichia coli*, its purification and initial crystallization assays will be described elsewhere (Dura *et al.*, 2010 ▶). Na_3_[Eu(DPA)_3_].6H_2_O complex where DPA stands for pyridine-2,6-dicarboxylate (*e.g.* dipicolinate) was prepared using the procedure described by Tancrez *et al.* (2005 ▶).

### 2.2. Crystallization

Crystallization was performed by vapour diffusion using the hanging-drop method at 293 K. Native PhTET1-12s crystals (∼200 µm × 140 µm × 20 µm) were grown within three weeks by mixing 1.5 µl of 6.2 mg ml^−1^ protein solution and 1.5 µl of 20–22% PEG 3350 (or 20% PEG 2000 MME), 100 m*M* Tris-HCl buffer at pH 7.5 and 200 m*M* trimethylamine *N*-oxide reservoir solution. PhTET1-12s derivative crystals (∼150 µm × 150 µm × 30 µm) were obtained within three weeks by cocrystallization with [Eu(DPA)_3_]^3−^ by mixing 1.5 µl of 6.2 mg ml^−1^ protein solution, 1.5 µl of 220 m*M* Na_3_[Eu(DPA)_3_].6H_2_O solution and 1.5 µl of reservoir solution containing 23–24% PEG 3350 (or 23–26% PEG 2000 MME).

Prior to data collection, derivative crystals were cryo-cooled in liquid nitrogen using mother liquor containing 20% ethyl­ene glycol as cryo-protectant.

### 2.3. Data collection and data processing

SAD data were collected on the FIP-BM30A beamline at the ESRF. Based on a fluorescence scan, the wavelength was chosen at the *L*_III_ europium absorption edge, and was set to 1.766 Å, which corresponds to the maximum value of *f*′′ (∼28 e^−^). Diffraction data were integrated using the program *XDS* (Kabsch, 2010*a* ▶,*b* ▶) and the integrated intensities were scaled and merged using the CCP4 programs *SCALA* and *TRUNCATE* (Collaborative Computational Project, Number 4, 1994 ▶). A summary of the processing statistics is given in Table 1 ▶.

## 3. Results

### 3.1. Derivative crystal form

As described in §2, we used crystallization conditions that led to a new high-resolution form of PhTET1-12s in space group *P*2_1_ with an entire dodecamer in the asymmetric unit (Dura *et al.*, 2010 ▶). Surprisingly, the addition of the tris-dipicolinate complex led to the initial *F*4_1_32 crystal form diffracting at low resolution, that was used for the initial structure determination of PhTET1-12s at 3.09 Å resolution (Porciero *et al.*, 2005 ▶; Schoehn *et al.*, 2006 ▶).

### 3.2. *De novo* structure determination

As shown in Table 1 ▶, the high value of *R*_ano_ clearly indicated the presence of tris-dipicolinate europium complex binding sites, which was then confirmed by the anomalous Patterson map. Despite the low resolution of the data, we attempted *de novo* phasing of the structure of PhTET1-12s. Using the program *SHELXD* (Sheldrick, 2008 ▶), we were able to locate one Eu site per TET-monomer. Heavy-atom refinement and initial phasing were performed using the program *SHARP* (La Fortelle & Bricogne, 1997 ▶). Phases from *SHARP* were improved by density modification using the CCP4 program *DM* (Cowtan & Main, 1996 ▶) assuming a solvent content of 50%.

### 3.3. Experimental 4.0 Å SAD phasing

**Figure 1:**
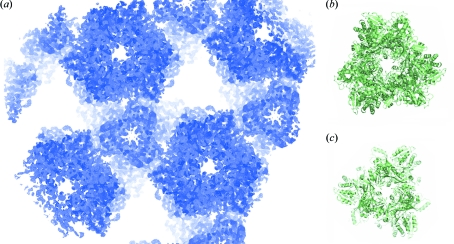
(*a*) Experimental 4.0 Å SAD electron density map contoured at 1.5σ showing (*b*) one of the large pores situated in each facet of the PhTET1-12s particle that gives access to the interior of the system and (*c*) one of the small orifices situated on each of the four apices. Figs. 1 ▶ and 2 ▶ were prepared using *COOT* (Emsley *et al.*, 2010 ▶) and *PyMOL* (http://www.pymol.org/).

**Figure 2:**
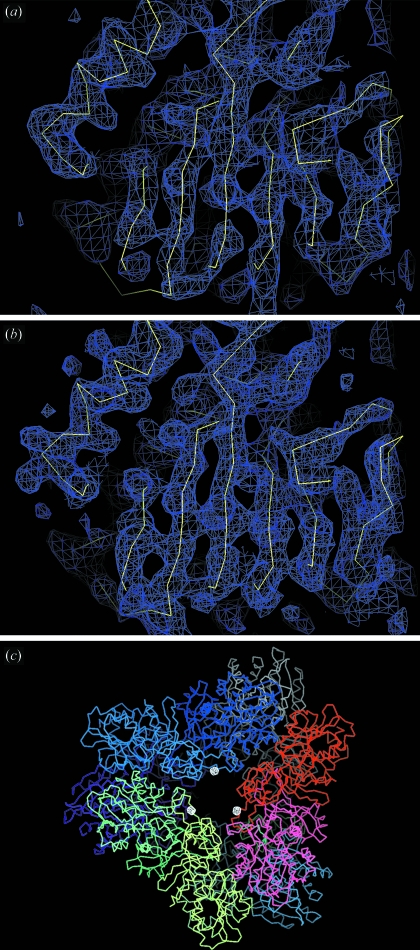
(*a*) Experimental 4.0 Å SAD electron density map. (*b*) Experimental 3.09 Å SIRAS electron density map. Both maps are contoured at 1.5σ. The model shown corresponds to PDB code 2cf4 (Schoehn *et al.*, 2006 ▶). (*c*) Anomalous Fourier map contoured at 10σ showing that the Eu^3+^ ion of the [Eu(DPA)_3_]^3−^ complex is located between two monomers on the large channel side of the particle. This dimer is considered as the minimal building block to form the whole TET1 particle.

Despite the low resolution, the experimental phases were accurate since the figure of merit after *SHARP* and *DM* are 0.369 and 0.731, respectively. The resulting experimental electron density map was of good quality (Fig. 1*a* ▶) since it allowed the polypeptide chain to be traced, as shown in Fig. 2(*a*) ▶. The overall shape of the PhTET1-12 subunit particle could be easily recognized with, on one side of the particle, the large channel (Fig. 1*b* ▶) assumed to be the entrance for the peptide substrate and, on the other side, the small channel (Fig. 1*c* ▶) assumed to be the exit pathway for the reaction products, which are individual amino acids.

### 3.4. Experimental 3.09 Å SIRAS phasing

We performed SIRAS (single isomorphous replacement with anomalous scattering) phasing using the 3.09 Å resolution native data set from which the structure of PhTET1-12s (PDB code 2cf4) was solved by molecular replacement (Rossmann, 1990 ▶). As for the SAD phasing, the SIRAS experimental phases were accurate since the figure of merit after *SHARP* and *DM* were 0.211 and 0.785, respectively. Despite the introduction of the tris-dipicolinate europium complex, the isomorphism between the native and derivative crystals was preserved. The resulting SIRAS electron density map was of high quality, as shown in Fig. 2(*b*) ▶.

## 4. Conclusion

We have shown that, using [Eu(DPA)_3_]^3−^, the high-phasing-power heavy-atom derivative of PhTET1-12s may be obtained by co-crystallization. Highly accurate experimental phases were obtained, even at the low resolution of this work (4.0 Å). The presence of the [Eu(DPA)_3_]^3−^ complex modified the crystal space group: from crystallization conditions that led to the monoclinic crystal form diffracting at high resolution, the introduction of [Eu(DPA)_3_]^3−^ induced the formation of cubic crystals. Pompidor *et al.* (2010 ▶) showed that the interaction between the protein and the [Ln(DPA)_3_]^3−^ complex occurs through hydrogen bonds between the O atom of the carboxylate groups of the DPA ligands and hydrogen-bond donor residues, and through hydrophobic π-stacking interaction between DPA rings and aromatic residues. In some cases this specific binding mode improves the protein–protein inter­action involved in crystal packing leading to supramolecular interactions. In the present structure it seems that it is not the case. Even if the low resolution of the data limits the modelling of the DPA ligand, the Eu^3+^ ion is located between two monomers on the large channel side of the particle, as shown in Fig. 2(*c*) ▶. These two monomers are supposed to be the minimal building block of the whole particle. Since the [Eu(DPA)_3_]^3−^ complex is bound within this building block, it did not directly influence the molecular packing as would be the case if bridging two building blocks. A possible explanation for the space group change is that binding of the tris-dipicolinate europium complex induces a small conformational change in the PhTET1-12s protomer, leading to the growth of the low-resolution crystal form.

The tris-dipicolinate europium complex binding site is located in the vicinity of a loop, which is assumed to be a key player in the addressing of the substrate toward the catalytic chambers of the TET particle (Durá *et al.*, 2009 ▶). To obtain new insights into this important functional zone, we therefore plan to attempt to increase the resolution of the experimental data either by soaking PhTET1 crystals in solutions containing [Eu(DPA)_3_]^3−^ or by preparing [Eu(DPA)_3_]^3−^ derivative crystals of PhTET2 or PhTET3, in order to obtain more precise experimental (*i.e.* model-bias free) information.

As mentioned, the binding of the lanthanoid complexes to the protein depends on their ligand, the non-covalent interaction being for example hydrophobic (for the complex Gd-HPDO3A; Girard, Stelter *et al.*, 2003 ▶) or through hydrogen bonding between arginine/lysine residues and the dipicolinate complex (Pompidor *et al.*, 2010 ▶). Thus, the probability of occurrence of the appropriate binding sites in the protein increases with the protein size. Combined with the strong anomalous signal of the lanthanoid ions, these complexes are thus efficient tools for solving the structure of large macromolecular assemblies, irrespective of their size.

## Figures and Tables

**Table 1:** Data collection and processing statistics of the [Eu(DPA)_3_]^3−^ derivative of TET1-12s

Space group	*F*4_1_32
Unit-cell parameter *a*	219.83 Å
Resolution range (high-resolution shell)	49.16–4.00 Å (4.22–4.00 Å)
No. of unique reflections	4207 (591)
*R*_merge_†	8.6% (22.3%)
*R*_pim_‡	3.0% (8.7%)
*R*_ano_§	6.8% (7.8%)
*I*/σ(*I*)¶	7.5 (3.5)
Completeness	99.9% (97.9%)
Multiplicity	13.7 (13.7)

†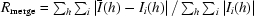 where 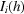 is the *i*th measurement of reflection *h* and 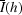 is the mean measurement of reflection *h*.

‡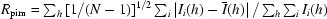. This indicator, which describes the precision of the averaged measurement, is most relevant (Weiss, 2001 ▶).

§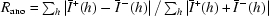 where 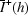 and 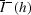 are the mean intensities of a Friedel mate.

¶ *I*/σ(*I*) is the signal-to-noise ratio for merged intensities.

## Footnotes

1 The different lanthanoid complexes can be purchased from NatX-ray (http://www.natx-ray.com/home.html).

## References

[bb1] Arnoux, P., Morosinotto, T., Saga, G., Bassi, R. & Pignol, D. (2009). *Plant Cell*, **21**, 2036–2044. 10.1105/tpc.109.068007 PMC2729612 19638474

[bb2] Bono, S. de, Riechmann, L., Girard, E., Williams, R. L. & Winter, G. (2005). *Proc. Natl. Acad. Sci. USA*, **102**, 1396–1401. 10.1073/pnas.0407298102 PMC547839 15671167

[bb3] Borissenko, L. & Groll, M. (2005). *J. Mol. Biol.* **346**, 1207–1219. 10.1016/j.jmb.2004.12.056 15713475

[bb4] Chaudhuri, B. N., Sawaya, M. R., Kim, C. Y., Waldo, G. S., Park, M. S., Terwilliger, T. C. & Yeates, T. O. (2003). *Structure*, **11**, 753–764. 10.1016/s0969-2126(03)00106-0 12842039

[bb5] Collaborative Computational Project, Number 4 (1994). *Acta Cryst.* D**50**, 760–763.

[bb6] Cowtan, K. D. & Main, P. (1996). *Acta Cryst.* D**52**, 43–48. 10.1107/S090744499500761X 15299724

[bb7] D’Aléo, A., Pompidor, G., Elena, B., Vicat, J., Baldeck, P. L., Toupet, L., Kahn, R., Andraud, C. & Maury, O. (2007). *ChemPhysChem*, **8**, 2125–2132. 10.1002/cphc.200700375 17847141

[bb8] Delfosse, V., Girard, E., Birck, C., Delmarcelle, M., Delarue, M., Poch, O., Schultz, P. & Mayer, C. (2009). *PLoS ONE*, **4**, e4712. 10.1371/journal.pone.0004712 PMC2651629 19266066

[bb9] Doublie, S. (1997). *Methods Enzymol.* **276**, 523. 9048379

[bb41] Durá, M. A. *et al.* (2010). In preparation.

[bb10] Durá, M. A., Rosenbaum, E., Larabi, A., Gabel, F., Vellieux, F. M. D. & Franzetti, B. (2009). *Mol. Microbiol.* **72**, 26–40. 10.1111/j.1365-2958.2009.06600.x 19291145

[bb11] Emsley, P., Lohkamp, B., Scott, W. G. & Cowtan, K. (2010). *Acta Cryst.* D**66**, 486–501. 10.1107/S0907444910007493 PMC2852313 20383002

[bb12] Franzetti, B., Schoehn, G., Hernandez, J.-F., Jaquinod, M., Ruigrok, R. W. H. & Zaccai, G. (2002). *EMBO J.* **21**, 2132–2138. 10.1093/emboj/21.9.2132 PMC125989 11980710

[bb13] Girard, É., Anelli, P. L., Vicat, J. & Kahn, R. (2003). *Acta Cryst.* D**59**, 1877–1880. 10.1107/s0907444903016871 14501144

[bb14] Girard, É., Chantalat, L., Vicat, J. & Kahn, R. (2002). *Acta Cryst.* D**58**, 1–9. 10.1107/s0907444901016444 11752774

[bb15] Girard, É., Stelter, M., Vicat, J. & Kahn, R. (2003). *Acta Cryst.* D**59**, 1914–1922. 10.1107/s0907444903020511 14573945

[bb16] Gras, S., Chaumont, V., Fernandez, B., Carpentier, P., Charrier-Savournin, F., Schmitt, S., Pineau, C., Flament, D., Hecker, A., Forterre, P., Armengaud, J. & Housset, D. (2007). *EMBO Rep.* **8**, 569–575. 10.1038/sj.embor.7400958 PMC2002535 17468740

[bb17] Hendrickson, W. A., Horton, J. R. & Lemaster, D. M. (1990). *EMBO J.* **9**, 1665–1672. 10.1002/j.1460-2075.1990.tb08287.x PMC551863 2184035

[bb18] Hermoso, J. A., Lagartera, L., González, A., Stelter, M., García, P., Martínez-Ripoll, M., García, J. L. & Menéndez, M. (2005). *Nat. Struct. Mol. Biol.* **12**, 533–538. 10.1038/nsmb940 15895092

[bb19] Jeudy, S., Stelter, M., Coutard, B., Kahn, R. & Abergel, C. (2005). *Acta Cryst.* F**61**, 848–851. 10.1107/S1744309105025856 PMC1978118 16511176

[bb20] Kabsch, W. (2010*a*). *Acta Cryst.* D**66**, 125–132. 10.1107/S0907444909047337 PMC2815665 20124692

[bb21] Kabsch, W. (2010*b*). *Acta Cryst.* D**66**, 133–144. 10.1107/S0907444909047374 PMC2815666 20124693

[bb22] Kahn, R., Fourme, R., Bosshard, R., Chiadmi, M., Risler, J., Dideberg, O. & Wery, J. (1985). *FEBS Lett.* **179**, 133–137. 10.1016/0014-5793(85)80207-6 3965297

[bb23] La Fortelle, E. de & Bricogne, G. (1997). *Methods Enzymol.* **276**, 472–494. 10.1016/S0076-6879(97)76073-7 27799110

[bb24] Márquez, J., Reinelt, S., Koch, B., Engelmann, R., Hengstenberg, W. & Scheffzek, K. (2006). *J. Biol. Chem.* **281**, 32508–32515. 10.1074/jbc.M513721200 16867985

[bb25] Molina, R., González, A., Stelter, M., Pérez-Dorado, I., Kahn, R., Morales, M., Moscoso, M., Campuzano, S., Campillo, N. E., Mobashery, S., García, J. L., García, P. & Hermoso, J. A. (2009). *EMBO Rep.* **10**, 246–251. 10.1038/embor.2008.245 PMC2658566 19165143

[bb26] Nagem, R. A. P., Dauter, Z. & Polikarpov, I. (2001). *Acta Cryst.* D**57**, 996–1002. 10.1107/s0907444901007260 11418768

[bb27] Pérez-Dorado, I., González, A., Morales, M., Sanles, R., Striker, W., Vollmer, W., Mobashery, S., García, J. L., Martínez-Ripoll, M., García, P. & Hermoso, J. A. (2010). *Nat. Struct. Mol. Biol.* **17**, 576–581. 10.1038/nsmb.1817 PMC6902435 20400948

[bb28] Pérez-Dorado, I., Sanles, R., González, A., García, P., García, J. L., Martínez-Ripoll, M. & Hermoso, J. A. (2010). *Acta Cryst.* F**66**, 448–451. 10.1107/S1744309110006081 PMC2852341 20383019

[bb29] Pompidor, G., D’Aléo, A., Vicat, J., Toupet, L., Giraud, N., Kahn, R. & Maury, O. (2008). *Angew. Chem. Int. Ed.* **47**, 3388–3391. 10.1002/anie.200704683 18350532

[bb30] Pompidor, G., Maury, O., Vicat, J. & Kahn, R. (2010). *Acta Cryst.* D**66**, 762–769. 10.1107/S0907444910010954 20606256

[bb31] Porciero, S., Receveur-Bréchot, V., Mori, K., Franzetti, B. & Roussel, A. (2005). *Acta Cryst.* F**61**, 239–242. 10.1107/S1744309105001910 PMC1952244 16511005

[bb32] Purdy, M. D., Ge, P., Chen, J., Selvin, P. R. & Wiener, M. C. (2002). *Acta Cryst.* D**58**, 1111–1117. 10.1107/s0907444902006509 12077430

[bb33] Rossmann, M. G. (1990). *Acta Cryst.* A**46**, 73–82. 10.1107/s0108767389009815 2180438

[bb34] Russo, S. & Baumann, U. (2004). *J. Biol. Chem.* **279**, 51275–51281. 10.1074/jbc.M409455200 15375159

[bb35] Schoehn, G., Vellieux, F. M. D., Asunción Durá, M., Receveur-Bréchot, V., Fabry, C. M. S., Ruigrok, R. W. H., Ebel, C., Roussel, A. & Franzetti, B. (2006). *J. Biol. Chem.* **281**, 36327–36337. 10.1074/jbc.M604417200 16973604

[bb36] Sheldrick, G. M. (2008). *Acta Cryst.* A**64**, 112–122. 10.1107/S0108767307043930 18156677

[bb37] Silvaggi, N. R., Martin, L. J., Schwalbe, H., Imperiali, B. & Allen, K. N. (2007). *J. Am. Chem. Soc.* **129**, 7114–7120. 10.1021/ja070481n 17497863

[bb38] Tancrez, N., Feuvrie, C., Ledoux, I., Zyss, J., Toupet, L., Le Bozec, H. & Maury, O. (2005). *J. Am. Chem. Soc.* **127**, 13474–13475. 10.1021/ja054065j 16190692

[bb39] Weis, W. I., Kahn, R., Fourme, R., Drickamer, K. & Hendrickson, W. A. (1991). *Science*, **254**, 1608–1615. 10.1126/science.1721241 1721241

[bb40] Weiss, M. S. (2001). *J. Appl. Cryst.* **34**, 130–135.

